# Food allergy risks and dining industry – an assessment and a path forward

**DOI:** 10.3389/falgy.2023.1060932

**Published:** 2023-03-29

**Authors:** Gabriel A. Stankovich, Christopher M. Warren, Ruchi Gupta, Sayantani B. Sindher, R. Sharon Chinthrajah, Kari C. Nadeau

**Affiliations:** ^1^ Department of Medicine, Sean N. Parker Center for Allergy and Asthma Research at Stanford University, Stanford, CA, United States; ^2^ Center for Food Allergy and Asthma Research at Northwestern University Feinberg School of Medicine, Chicago, IL, United States; ^3^ Department of Environmental Health, Harvard T.H. Chan School of Public Health, Boston, MA, United States

**Keywords:** food allergy, food label, restaurant, allergy prevention, precautionary labeling, quality of life

## Abstract

Food allergies have increased in prevalence over the last few decades and continue to grow. Consumption of even trace amounts of common foods can cause a rapid allergic reaction (generally within minutes) which can be mild to severe to even life-threatening. Eating at restaurants poses a risk of allergic reactions for those with food allergies due to inadequate, inconsistent labeling of allergens in foods. Here, we review food labeling rules and practices in the restaurant industry and compare and contrast it with food labeling for prepackaged foods. We review global and United States trends, and provide a brief historical overview. The paper describes the key legal and economic motivations behind restaurant food labeling. Next, we describe novel risk-driven policies and new biotechnologies that have the potential to change food labeling practices worldwide. Finally, we outline desirable federal regulations and voluntary information disclosures that would positively impact the public health aspects of restaurant food labeling and improve the quality of life for people with severe food allergies.

## 1. Introduction

Food allergies are IgE-mediated allergic reactions to specific common food proteins. Consumption of even a minute amount of allergenic foods through accidental ingestion can cause a rapid allergic reaction involving one or more organs. Symptoms can be mild, moderate, severe, and even fatal (1). The most severe reactions, often involving multiple organs, are termed anaphylaxis.

**Figure 1:**
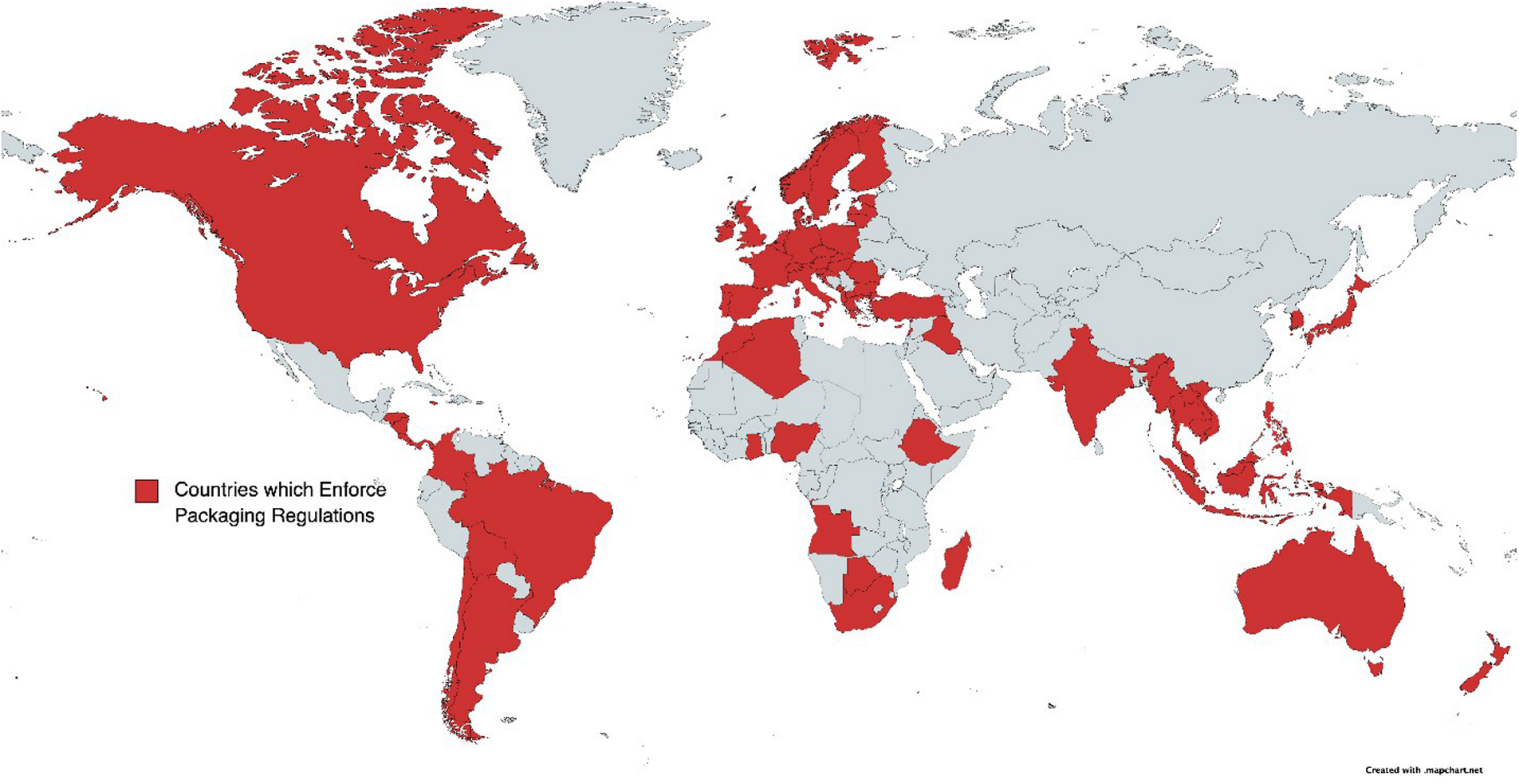
Countries which enforce allergen labeling regulations for packaged foods.

Food allergies in the United States affect an estimated 26 million adults and 6 million children (10.8% and 8%, respectively) (2–4). The corresponding medical costs are very significant – the healthcare burden includes direct costs of $4.3 billion annually in 2013 (5), and the cost per child per year was $4,184 (6). The total economic impact, which includes lost labor productivity values and opportunity costs, is much higher, estimated at $29.4 billion annually (5). Furthermore, the problem is growing – the total number of anaphylactic reactions increased by 377% from 2007 to 2016 (7). An increase of 615% has been observed in the United Kingdom from 1992 to 2012 (8). These observed increases in the burden of food allergy occur not only in the United States but are also reported elsewhere. For example, China reports countrywide food allergy prevalence among 1%–2% of adults and 5% of children (7, 9). European data from the recent EuroPrevall study show a wide variation among adults in different countries, ranging from 0.3% in Greece to 5.6% in Switzerland (10); similar variation was observed in children, ranging from 1.9% in Iceland to 5.6% n Poland (11). In the Chongquing metropolitan region in southwestern China, an increase in the incidence of food allergies from 3.5% to 7.7% was reported from 1999 to 2009 (7).

Most anaphylactic reactions occur outside of the home, with 25% occurring while dining at restaurants (12). Some estimate that 74% of all allergy-related food reactions involve non-pre-packaged food (13). For example, 59% of food-related anaphylaxis hospitalizations in the United Kingdom are attributed to catering establishments (8). An earlier set of case studies (14) with fatal outcomes produced a similar ratio of 5/7. Furthermore, a recent study reports that 53.9% of food allergy reactions at United States restaurants occurred despite the restaurant staff being notified (15). Without a targeted policy change, allergic reactions at restaurants are expected to increase further, as more and more Americans develop food allergies and consume foods outside of their homes. In 2019 (the last pre-pandemic year) Americans have spent more than half (54%) of their food dollars away from home, the highest level ever recorded (16). The spending on away-from-home food consumption as a portion of total food expenses has steadily increased at the rate of 5%–6% per decade since 1960 (12).

Around 30% of restaurant patrons self-identify as having a food allergy or sensitivity (13). Among people in the United States Peanut and Tree Nut Allergy Registry, 13.7% have experienced reactions in restaurants and food establishments (17). While required allergen disclosure methods at restaurants vary globally, the tourism industry strongly feels that customers with food allergies are likely to seek out food establishments with allergen content in their foods publicly disclosed, including a procedure for prevention of allergen cross-contamination (13).

Adolescents are a group that is particularly at risk of allergic reactions at restaurants (18). As many of them transition from high school to institutions of higher education, they can rely less on their parents or guardians and are more likely to self-manage their food allergies, increasing their risk of allergen exposure. In addition, adolescents take more risks than other age groups (19), eat food outside the home more often, are less likely to carry epinephrine auto-injector, and are more likely to eat food containing allergen precautionary or warning labels (20, 21). Notably, regulations covering institutions of higher education are particularly scarce despite the high prevalence of food allergies among this group and their frequent reliance on institutional dining facilities (18).

In this paper, we advocate a risk-based approach to policy development for restaurants that would combine realistic assessments of allergen exposure consequences and the likelihood of cross-contamination with science and data-driven metrics for monitoring *via* the implementation of additional federal regulation. Furthermore, we argue that the current regulatory regime is leading to suboptimal physical health and psychosocial outcomes in terms of greater anxiety/social limitation for people affected by food allergies and a greater number of accidental allergen exposures, anaphylactic events, hospitalizations, and even deaths. Finally, we outline some possible future directions and research issues that would help inform and guide desirable policy and regulatory outcomes.

## 2. Direct detection and quantification of low doses of allergens

There is an ongoing effort to define thresholds of allergen presence in processed food that would inform the need for precautionary allergen labels (PAL). These labels are voluntary and not common in the restaurant industry today. The thresholds are allergen-dependent and in the low mg range for several allergens (22). A recent position paper suggested a universal threshold of 0.5 mg per 100g as universal guidance for food labeling (23). Similar thresholds for total protein amounts (e.g., 2 mg for peanuts) have been established as reference doses in a recent FAO report (45). Among qualitative methods, immunoassay methods known as qualitative lateral flow strips (LFSs) are widely used by the food industry to assess the cleanliness of shared equipment after cleaning. While qualitative, LFSs are specific and sensitive down to the range of 5 ppm in the swab extract (5). They are well-suited for the restaurant industry, as they provide near-instantaneous results. The process of developing and validating LFSs for widely different uses is still ongoing and has great promise for applications in both food processing and restaurant industries.

The key quantitative method for the detection of food allergen residues is the enzyme-linked immunosorbent assay (ELISA), which has been widely commercialized in the food industry (5), but requires an on-site laboratory facility. New technologies such as proteomics have profoundly advanced the detection and quantification of allergens (5). While not deployable today, we expect their maturation to be aided by food processing and restaurant industry demand.

## 3. An overview of food allergy regulations for prepackaged foods

International organizations have spurred food allergy regulations in many countries. In 1963, the World Health Organization (WHO) and the Food and Agricultural Organization (FAO) collaborated to form the Codex Alimentarius Commission. The main concern of the commission was to protect the health of consumers and promote fair practices in the food industry while establishing food safety standards (24). The Commission turned its attention to allergens as a health concern in 1999. This recognition by the international community that food allergies pose an essential public health issue necessitating the need to protect customers resulted in guidelines with wide-ranging consequences, such as Directive 2003/89/EC by the European Parliament in 2003 (24), later consolidated as Regulation 1169/2011 (25). This document invokes previous legal documents defining, for example, "food business" and "food additive", and proceeds to provide a detailed list of mandatory food information (25) as well as the compulsory placement of this information.

In the United States, food allergen regulations stem from several sources. Food, Drugs, and Cosmetics Act (FDCA) was introduced in 1938 and amended in 1958. Among other provisions, it gave FDA authority over prepackaged foods in interstate commerce.. The 2004 Food Labeling and Consumer Protection Act (FALCPA) (129) is the most relevant code for allergen labeling. It covers eight "major food allergens"—milk, egg, fish, crustacean shellfish, tree nuts, wheat, peanuts, and soybeans—and food ingredients containing proteins derived from any one of the specified foods. FALCPA requires that the food allergen information be provided on the label. Sesame was added as the ninth allergen in the 2021 Food Allergy Safety, Treatment, Education, and Research (FASTER) act which became effective on January 1, 2023 (46). Just like the previous rulings, this act does not apply to restaurants.

The complexity and heterogeneity associated with allergy regulations are not unique to the United States, as many countries as well as the European Union (EU), are in a similar position (26) (See Figure 1). For example, China implemented its first allergen labeling laws in 2012 for domestically produced foods (27). Their National Standard has two parts to their labeling requirements (7, 28): General Rule for the Labeling of Prepackaged Foods, and General Rule for Nutrition Labeling of Prepackaged Foods. The Standard uses the same list of key allergens as United States regulations, and is applied only to prepackaged foods. It does not apply to foods sold for immediate consumption, as enforced in the EU, nor does it apply to large manufacturers providing food to caterers, as prescribed, for example, in Australia and New Zealand. An updated version of General Rule that includes imported foods has been opened for comments in 2019, but its implementation has been delayed (29), possibly due to the Covid-19 pandemic.

The food industry is keenly aware of the impact of food labeling regulations. In an industry-wide survey (32), cleaning procedures, employee training, and the potential for a recall due to allergen cross-contamination were most frequently rated as the critical factors in food allergen management. In terms of expenses, recalls ranked first, followed by cleaning procedures. Although a large majority (96%) of participating companies had allergen control plans in place, nearly half (42%) had experienced a recall in the preceding five years. The industry sees precautionary allergen labeling as a means to communicate better with consumers (32) and possibly reduce liability.

## 4. Regulation for restaurants

Despite many similarities, the prepackaged foods industry has numerous advantages in allergen management over the restaurant industry. Typical manufacturing facilities are large, long processing runs are made of many products, and the necessary time is available for cleaning shared equipment and facilities. A retail food establishment could make dozens of different dishes in a very limited space with shared equipment and utensils during compressed meal preparation times, so the opportunities for errors are abundant. In addition, employee turnover tends to be significantly higher in food service compared to the prepackaged foods industry, so employee training becomes a more formidable challenge.

Unsurprisingly, the codes for restaurants are less developed than those applicable to prepackaged food. In the United States, the FDA does not regulate retail food establishments which fall under state and local authorities. FALCPA directs the Secretary of Health and Human Services to "pursue revision of the Food Code to provide guidelines for preparing allergen-free foods in food establishments, including in restaurants, grocery store delicatessens, and bakeries." FALCPA is specifically preemptive, preventing other governmental entities (such as those at the state or local level) from adopting labeling requirements that are different from those in FALCPA.

**Figure 2:**
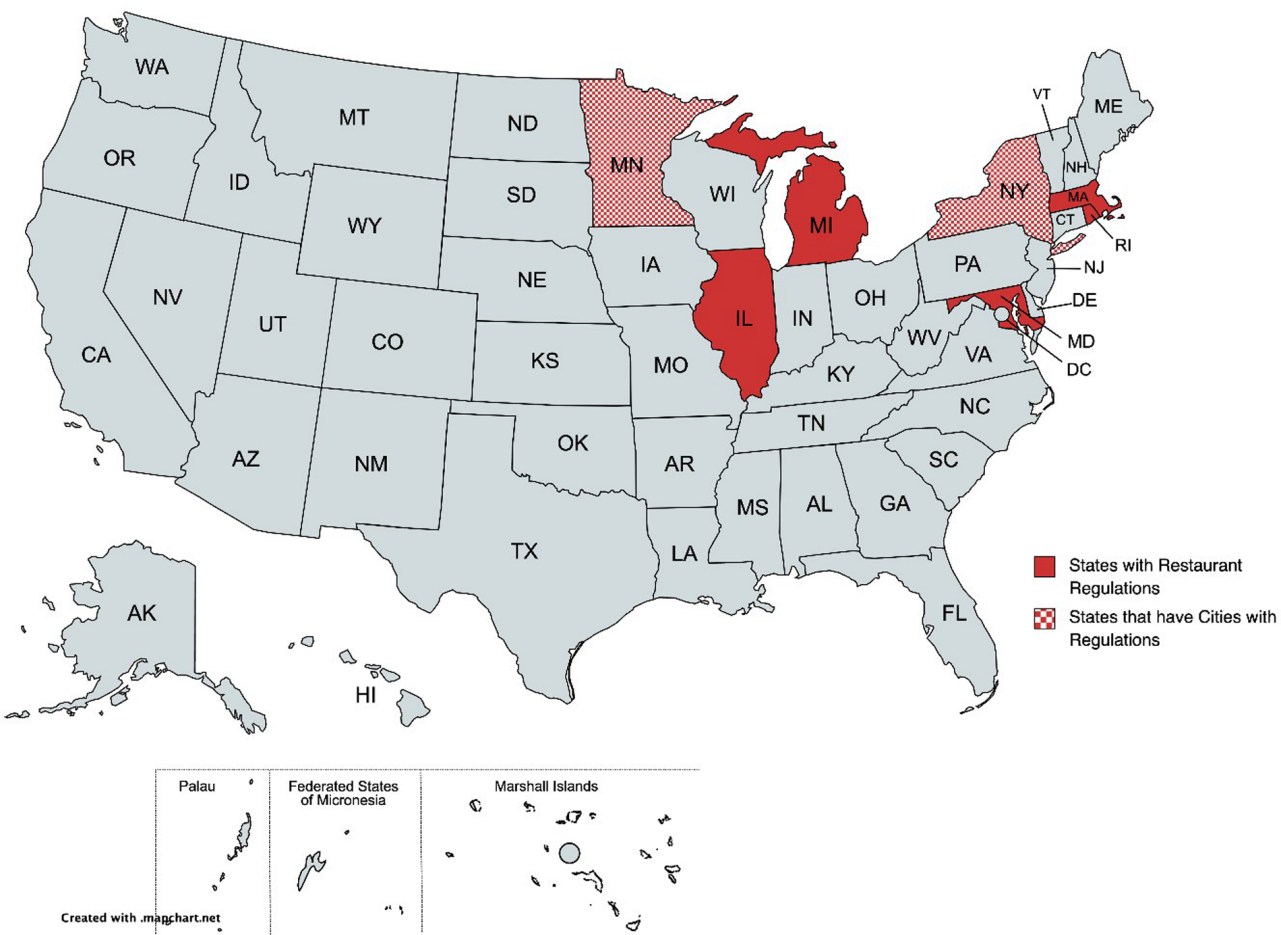
States with restaurant allergen labeling regulations.

Another source of guidelines is the Food Code which is published by the Public Health Service and FDA. The Food Code originated in 1906 and has been revised many times (the last time in 2017 with the intended 4–year revision policy). The 2005 Food Code, published the year after FALCPA, discusses the management of food allergens in more detail than previous editions of the code (12) and refers to specific labeling requirements in FALCPA. Local, state, tribal, and federal regulators use the FDA Food Code as a model to develop or update their own food safety rules. Since for example, state regulators refer to a specific version of the FDA Food Code in their own documents, there exists some heterogeneity in terms of adoption and implementation of the Code across the United States. Figure 2 illustrates the resulting heterogeneity in regulations in different United States states. Code enforcement is left to state and local authorities, resulting in variations across the country.

For example, the FDA has stated that FALCPA's labeling requirements "do not apply to foods provided by a retail food establishment if they are placed in a wrapper or container in response to a consumer's order—such as the paper or box used to convey a sandwich that has been prepared in response to a consumer's order" (12). FALCPA, however, does apply to bulk food sold to restaurants, and the information from food labels could be transferred to menus. In practice, however, the complexities of restaurant operations often preclude this flow (30).

The Food Code has largely been consistent with FALCPA, as many foods in restaurants are excluded from labeling requirements, as FDA has defined "packaged" to exclude "a wrapper, carry-out box, or other nondurable container used to containerize food with the purpose of facilitating food protection during service and receipt of the food by the consumer." The Food Code, does, however, attempt to add a dose of clarity to the allergen handling expectations for restaurants. It noted that food packaged in a food establishment must be properly labeled for major food allergens. It also stated that a person in charge must ensure that "employees are properly trained in food safety, including food allergy awareness, as it relates to their assigned duties" (12).

The Affordable Care Act (ACA) of 2010, also known colloquially as Obamacare, introduced Section 4205, which amended the FDCA to require nutrition labeling of standard menu items at chain restaurants (12, 31). While primarily concerned with obesity as a public health concern, this Act also impacts food labeling in general.

The allergy labeling-related Federal Acts like FALCPA and the Food Code both aim to promote public health by informing consumers about food allergens when they are present in regulated food products. However, their public health utility depends upon their acceptance and enforcement by the state and local authorities, leading to delayed and heterogeneous implementation.

## 5. Legal aspects of food allergy regulation in restaurants

Case law on whether a severe food allergy may constitute a disability under the Americans with Disabilities Act (ADA) of 2008 is limited (12), as the issue has not been litigated in federal courts. There exists, however, a wide agreement that the answer is likely to be in the affirmative, as indicated by the 2012 agreement between the United States Department of Justice and Lesley University, which recognized that "food allergies may constitute a disability under the ADA.'

A person injured by an allergic reaction to food served in a restaurant has two key options in a legal case. The first is called the failure to warn claim, in which the plaintiff would have to prove that the restaurant failed to provide a reasonable warning and that failure rendered the food unsafe (13). The second is called a manufacturing defect claim, as in the case of cross-contamination with an allergen that was not supposed to be in the food. The second path is sometimes perceived as easier for the plaintiff but it still poses challenges to prove that the food was defective, that it was defective when it left the control of the restaurant, and that the defect caused the allergic reaction (13).

Section 4205 of the Affordable Care Act ("Obamacare") brought a new requirement for nutritional labeling of menus at chain restaurants (20 or more locations under the same name). It focuses on the caloric information for standard menu items, intending to address the obesity problem. Since the Act is quite cryptic about the scope of this Section, it necessitated the later FDA ruling defining a "restaurant or similar retail establishment" as "a retail establishment that offers for sale restaurant-type food, except if it is a school." It is easy to imagine an amendment to Section 4205 covering allergy-related labeling in restaurants.

## 6. Economic aspects of food allergy regulation in restaurants

A key aspect affecting the economics of restaurants is that information asymmetry exists between the restaurant and a potential customer. Because of the lack of information about the restaurant's adherence to safe food preparation, a customer may decide to dine less often or forgo the restaurant's visit altogether (13). It is well appreciated in the economics community (13) that information asymmetries can lead to inefficient markets or even complete market failure. Therefore, a goal for the government would be to intervene and reduce or eliminate this asymmetry by measures such as mandatory labeling, standards, and educational efforts.

The educational efforts can also be led by individual companies, their industry associations, or vendors. For example (18), the National Restaurant Association's ServSafe is a 1.5- to 2-hour online course that addresses a host of practical issues such as defining food allergens, recognizing symptoms, identifying allergens, the dangers of allergen cross-contact, proper cleaning methods, proper communication, clean workstations and self-serve areas, special dietary requests, dealing with emergencies, the importance of food labels, handling food deliveries, proper food preparation, and cleaning and personal hygiene.

A recent study in a mature hospitality market in Croatia (13) revealed that only 2.1% of restaurant websites disclose allergen information, and only a small portion (6.5%) of those disclose specific allergens. In contrast, 24.6% of social media reviews of restaurants in the area included comments about food allergens; however, these reviews received very few responses from the restaurants. While published studies are sparse, this situation is similar to the assessment of United States restaurants in 2009 (33) or food establishments in Switzerland in 2022 (34). An implication is that restaurants globally need to take the initiative in becoming a partner in forming the overall dining narrative for allergy-affected customers while potentially reaching a broader set of potential customers.

It is also widely appreciated in the hospitality industry that customers with allergies tend to be loyal to restaurants that provide allergen-free foods, often bringing larger groups of diners without allergy concerns. This, in turn, can boost profits for the industry, which is notorious for low-profit margins (13). For example, Disney branded parks experienced remarkable growth in sales of allergy-friendly meals (referred to as "special" in (13, 35)) once they started to actively promote their expertise in allergy-friendly dining (Table 1, data from (13, 35)).

**Table 1:** Special dietary meals served in disney-branded parks in the United States.

Year	Special Meals Served	Location
2005	52,000	Disney World
2009	138,000	Disneyland
2009	192,000	Disney World
2012	625,000	Disney World and Disneyland

## 7. Examples of proactive rule making in states, cities, and industry sectors

In 2009, the Commonwealth of Massachusetts enacted the Food Allergy Awareness Act (FAAA), and thus became the first state to pass a law related to food allergen awareness in restaurants (12). The Act stipulates that "a person licensed as an innholder or common victualler, when serving food" (1) post an approved food allergy awareness poster in the staff work area, (2) include a notice informing customers of their "obligation to inform the server about any food allergies," and (3) requires "[a] person in charge and certified food protection manager" to view a video concerning food allergies as part of a course to obtain certification as an approved food protection manager. To assuage concerns about possible overlaps with other existing regulations, the FAAA states that except as expressly provided, it does not create or change a private cause of action or change the duty under any other statute or the common law (12). While being a step in the right direction, the FAAA stops short of requiring that food establishments provide ingredient or allergen information for menu items. It creates potential remedies (the allergy awareness poster and certification for staff) without mandating that the restaurant staff take specific steps to prevent cross-contamination of food.

The restaurant menu labeling movement initially concerned obesity as a public health problem (12). The most important legal development in this regard occurred in New York City. The first attempt at regulation in 2006 was spear-headed by the NYC Board of Health and it required "some restaurants [to] post calorie information on menus and menu boards." (12). The New York State Restaurant Association (NYSRA) promptly sued the Board of Health and the United States District Court for the Southern District of New York ruled in 2007 that the regulation was preempted by federal law, referring to the Nutritional Labeling and Education Act of 1990 (NLEA) (12). The Board of Health then introduced a new regulation in January of 2008, requiring that covered establishments "[display] calorie information… on all menu boards and menus, as well as on food item display tags, adjacent or in close proximity, to the menu item, using a font and format that is at least as prominent in size as that used to post either the name or price of the menu item" (12). An important difference with respect to the previous attempt was that 2008 regulation was aimed at chain restaurants and defined the covered establishment as "a food service establishment within the City of New York that is one of a group of 15 or more food service establishments doing business nationally, offering for sale substantially the same menu items, in servings that are standardized for portion size and content, that operate under common ownership or control, or as franchised outlets of a parent business, or do business under the same name". The decision by the United States Court of Appeals for the Second Circuit concluded that the 2008 NYC menu labeling rule was not preempted by federal labeling law and did not violate the First Amendment of the United States Constitution (12).

This ruling has opened the path for cities, counties, states, and, ultimately, the federal government to enact menu labeling requirements. For example, California became the first state to pass such a regulation in 2008. As another example, Santa Clara County regulations cover chain restaurants in the county's unincorporated area, define the covered establishments as those belonging to a chain with fourteen or more restaurants in California, and require that nutritional information be provided (12).

The airlines represent an industry sector of great relevance to people with food allergies, primarily because of limited mobility and food choices during flights (18). Given the current practice of serving prepackaged food, it is surprising that in the United States caterers who provide such items are not subject to FALCPA labeling requirements unless they distribute food that was packaged and sold in interstate commerce. Even this requirement is not being enforced at this time (18). In contrast, EU Allergen Legislation Regulation No.1169/2011 on The Provision of Food Information to Consumers (13) requires complete labeling information for prepacked food, including allergens and a quantitative indication of ingredients. For example, Article 22 states that quantitative indication is needed "where the ingredient or category of ingredients concerned:

a) appears in the name of the food or is usually associated with that name by the consumer;
b) is emphasized on the labeling in words, pictures, or graphics; or
c) is essential to characterize a food and to distinguish it from products with which it might be confused because of its name or appearance." (25)

This regulation is wide-ranging as it is pertinent "to all foods intended for the final consumer, including foods delivered by mass caterers" and applies to "catering services provided by transport undertakings when the departure takes place on the territories of the Member States to which the Treaties apply." (18).

## 8. VITAL, HACCP, and HARPC

The Voluntary Incidental Trace Allergen Labelling (VITAL), Hazard Analysis and Critical Control Point (HACCP), and Hazard Analysis and Risk-Based Preventive Controls (HARPC) are examples of a new generation of labeling tools that add two essential dimensions - quantification and risk analysis.

Pioneered by the Allergy Bureau of Australia and New Zealand, VITAL (36) is based on two quantities – the reference dose for the allergen and the reference amount specific to the food. The reference doses are derived from confirmatory oral food challenge data and signify the level of allergenic protein exposure to which only the most sensitive 1% of the allergic population will likely experience the adverse reaction (i.e., ED01). The reference amount is the maximum amount of food eaten on a typical eating occasion. Depending on the outcome of comparing the reference dose with the reference amount, the recommendation may be that no precautionary statement is warranted (Action Level 1) or that such a statement is required (Action Level 2). The VITAL procedure is voluntary and versatile as it allows the study of possible food cross-contamination in industrial facilities. It has been updated several times, and the current version, 3.0, was released in 2019 (37). However, reference doses for allergenic foods have only recently been established internationally (45), and existing national regulations tend to vary widely (38).

VITAL naturally fits in the HACCP- and HARPC-based allergen control plan that ensures that allergens are appropriately labeled (12, 39). HACCP has been primarily oriented toward food safety regulations by the FDA (40, 41). Its seven fundamental principles include: "hazard analysis, critical control point (CCP) identification, establishing critical limits, monitoring procedures, corrective actions, verification procedures, and record-keeping and documentation. Under such systems, if a deviation occurs indicating that control has been lost, the deviation is detected and appropriate steps are taken to reestablish control in a timely manner to assure that potentially hazardous products do not reach the consumer." (40) In the case of the EU, general rules for the control of hazards are defined in Regulation (EC) No. 852/2004 (42), which covers the hygiene of foodstuffs. It fully supports the HACCP and states that "the HACCP system should not be regarded as a method of self-regulation and should not replace official controls." Most importantly, the HACCP approach is now voluntary at the retail level in many countries (12, 39). HARPC, on the other hand, does not require CCPs, as it aims to enforce preventive controls that identify potential food supply risks and implement appropriate corrective actions proactively to prevent contamination. Adherence to HARPC has been a legal obligation of food manufacturers since the passage of the Food Safety Modernization Act (FSMA) in 2011, and it is enforced by FDA (34). However, incomplete documentation about preventive control guidance in FSMA limits the overall acceptance of HARPC in food packaging industry.

The restaurant industry is well behind the prepackaged food industry in terms of allergen management. For example, applying HACCP to the restaurant industry is likely be more challenging because of the need to identify and monitor several CCPs, e.g., for cross-contamination (33, 43, 44).

## 9. Discussion: summary and a way forward

The regulatory environment around food allergen labeling of restaurant menus is at a stage similar to the labeling of prepackaged food before FALCPA or nutritional labeling before the Nutritional Labeling & Education Act of 1990. While food processing and the restaurant industry are interrelated, Federal legislation has treated them as separate entities since at least the FD&C Act of 1938. With the growth of the restaurant industry, appropriate steps must be taken to alert consumers of common food allergens on menus. In the United States, additional federal regulations are needed to move in this direction.

An argument can be made that the FDA already has the authority to advance regulations requiring food allergen labeling and management in restaurants under the current law. FDA has jurisdiction over "food," which the FDCA defines, in part, as "articles used for food or drink for man" and "articles used for components of any such article" (13). It would also resolve the current legal inconsistency that the availability of key information on major food allergens rests on whether or not a food has been prepackaged. However, a distinct legislative effort would likely be needed for the FDA to take on this expanded role.

State and local regulations need to be aligned with the latest versions of the Food Code and kept abreast of the evolving technological means to check compliance. The food industry can help this transition by fully adopting risk-based methodologies like VITAL together with proteomics-based detection and quantification tools. Although the Food Code incorporates HACCP/HARPC principles and identifies allergens as hazards, it would be necessary for the restaurant and hospitality industries to embrace the HARPC approach to improving food safety. HARPC is systematic and rational, leading to an increase in customer confidence and in focus and ownership of allergen safety. Some of the HARPC steps will likely help restaurants economically by broadening their customer base and building loyalty while helping with liability protection. At the same time, comprehensive HARPC compliance is likely costly and could prove challenging for smaller restaurants. This is where governmental agencies like FDA could help, for example by producing food allergen management manuals for owners and operators of restaurants to parallel (41). Activities like maintaining up-to-date online menus with complete allergy information and maintaining allergy-related information on social networks come with small costs to the restaurants yet present increased economic potential.

A comprehensive solution requires all significant actors to make substantial efforts to participate and cooperate in implementing and monitoring regulatory changes. These undertakings are necessary for enabling a safer environment and better quality of life for people with food allergies. The federal government's role is essential in developing new policies and regulations to dramatically improve health and social outcomes for the growing number of people with food allergies.

States should adopt regulations for restaurant allergen menu labeling, and harmonize them with Federal legislation as it materializes. The scientific and medical communities have a pivotal role in building and analyzing the data sets (9) that would serve as a basis for developing and informing such governmental policies. Specifically, research on best practices for allergen control and management in restaurant and food service facilities is needed, together with improved training for restaurant management and staff, for example, by expanding the breadth and depth of the ServSafe program.
